# P-1993. Investigating Perspectives of Next-generation Sequencing Infectious Diseases Diagnostic Testing

**DOI:** 10.1093/ofid/ofaf695.2158

**Published:** 2026-01-11

**Authors:** John M Flores, Eric Roessler, Jose Paredes Sosa, Josie Majowka, Kaitlyn Gomez, Daniel Z P Friedman

**Affiliations:** Cook County Health / University of Chicago, Chicago, IL; University of Chicago, Chicago, Illinois; Advocate Masonic Illinois, Chicago, Illinois; University of Chicago Medicine, Chicago, Illinois; University of Chicago, Chicago, Illinois; University of Chicago, Chicago, Illinois

## Abstract

**Background:**

Infection represents a significant health burden affecting all patient demographics, and thousands of pathogens are known to cause infections in humans; however, many pathogens may not be identified using traditional diagnostic methods. This limitation arises partly from the challenges of growing organisms in conventional culture media, and when employing PCR, the requirement for sequence-specific amplification complicates matters. To overcome these obstacles, in recent years, plasma cell-free metagenomic next-generation sequencing (NGS) has emerged as a noninvasive method to identify and quantify pathogen DNA in plasma. We aimed to gather insights on the clinical use of NGS from both Infectious Diseases (ID) and Non-ID providers.

**Table 1:**
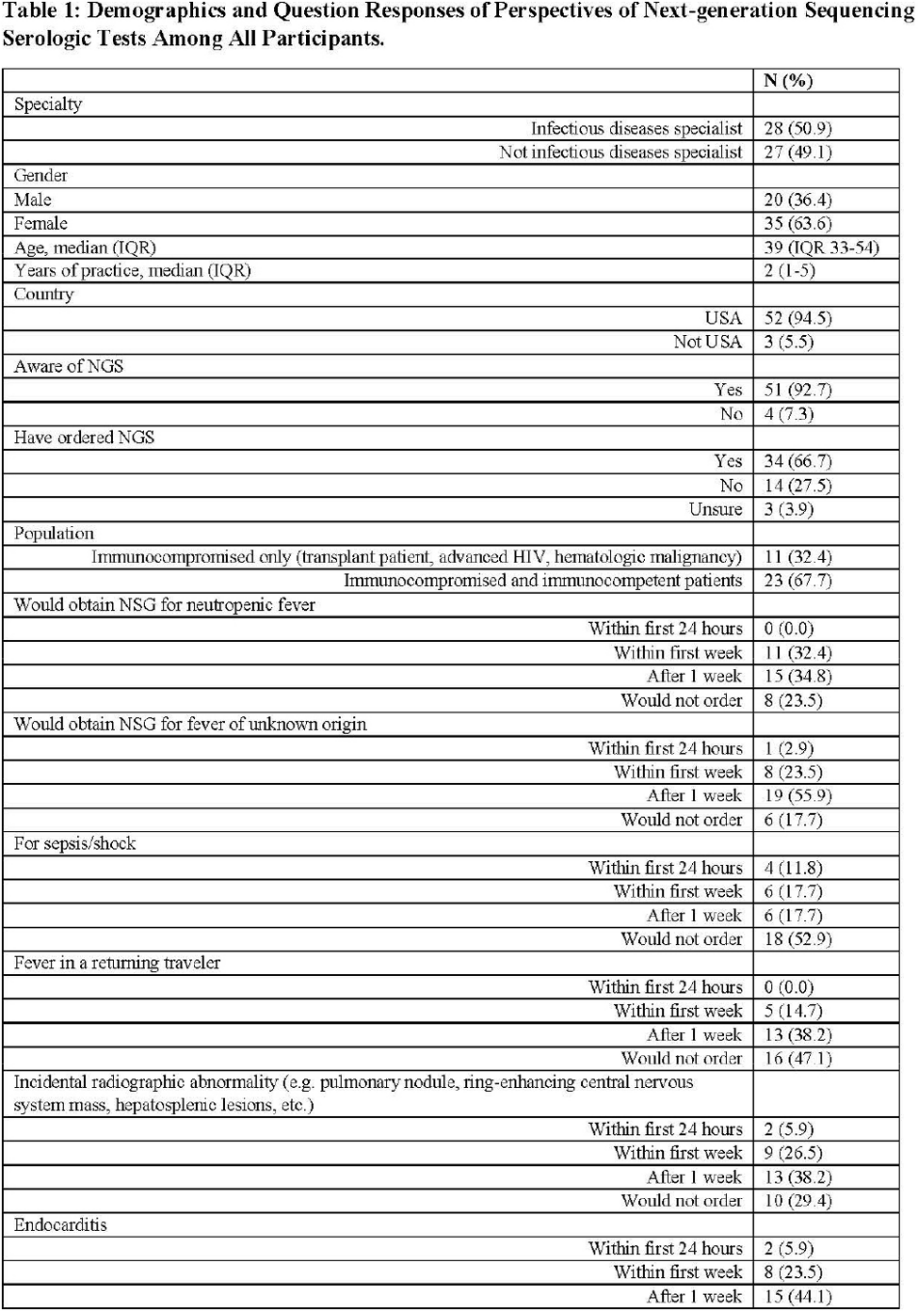
Demographics and Question Responses of Perspectives of Next-generation Sequencing Serologic Tests Among All Participants

Page 1

**Table 1:**
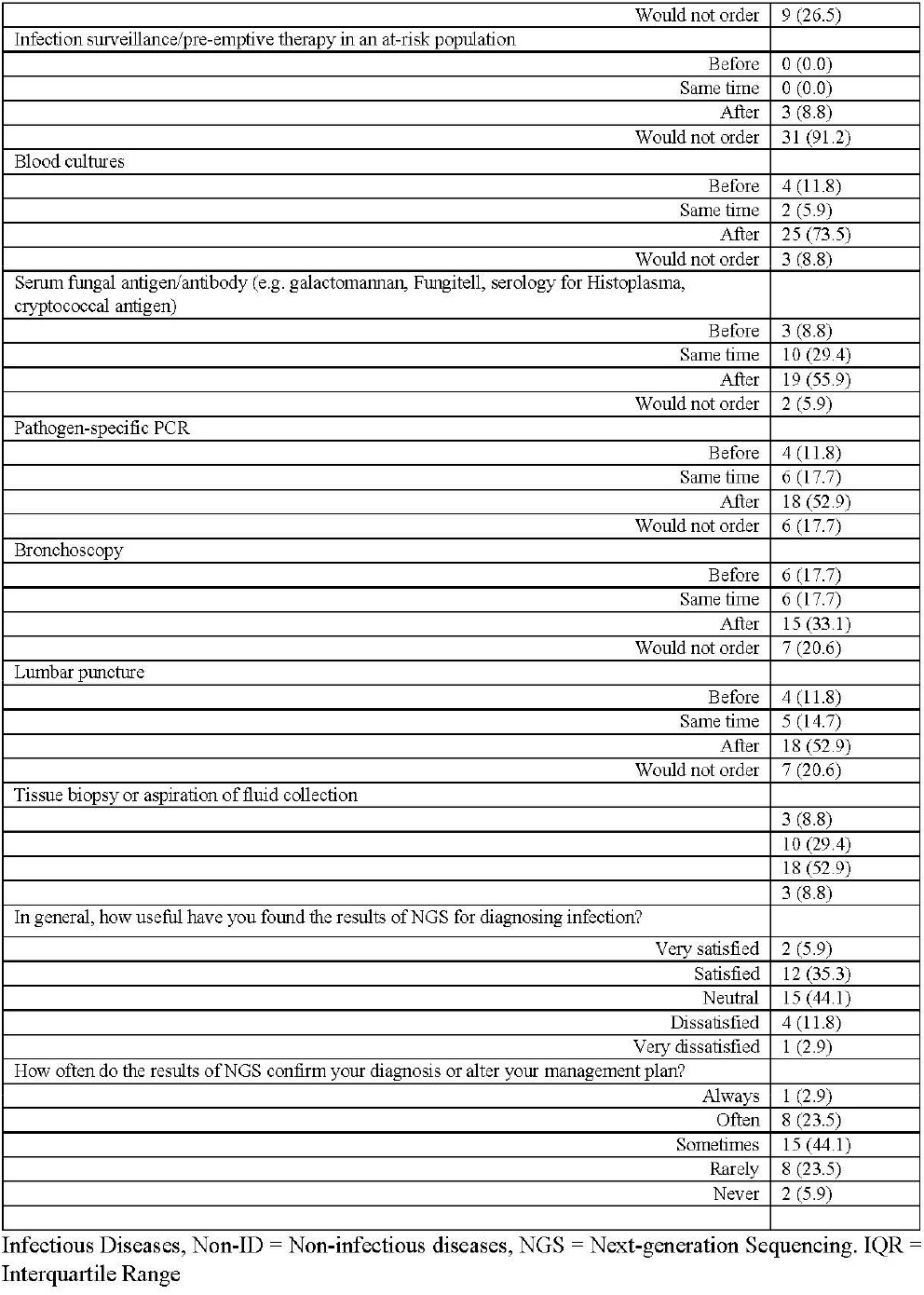
Demographics and Question Responses of Perspectives of Next-generation Sequencing Serologic Tests Among All Participants

Page 2

**Methods:**

From October 1-November 30, 2024, we conducted a cross-sectional survey among USA and Canadian providers using a purpose-driven sampling among national ID and internal medicine society membership databases. Questions related to NGS use in specific scenarios (Supplemental Document 1). 6 weekly email reminders were sent.

**Table 2:**
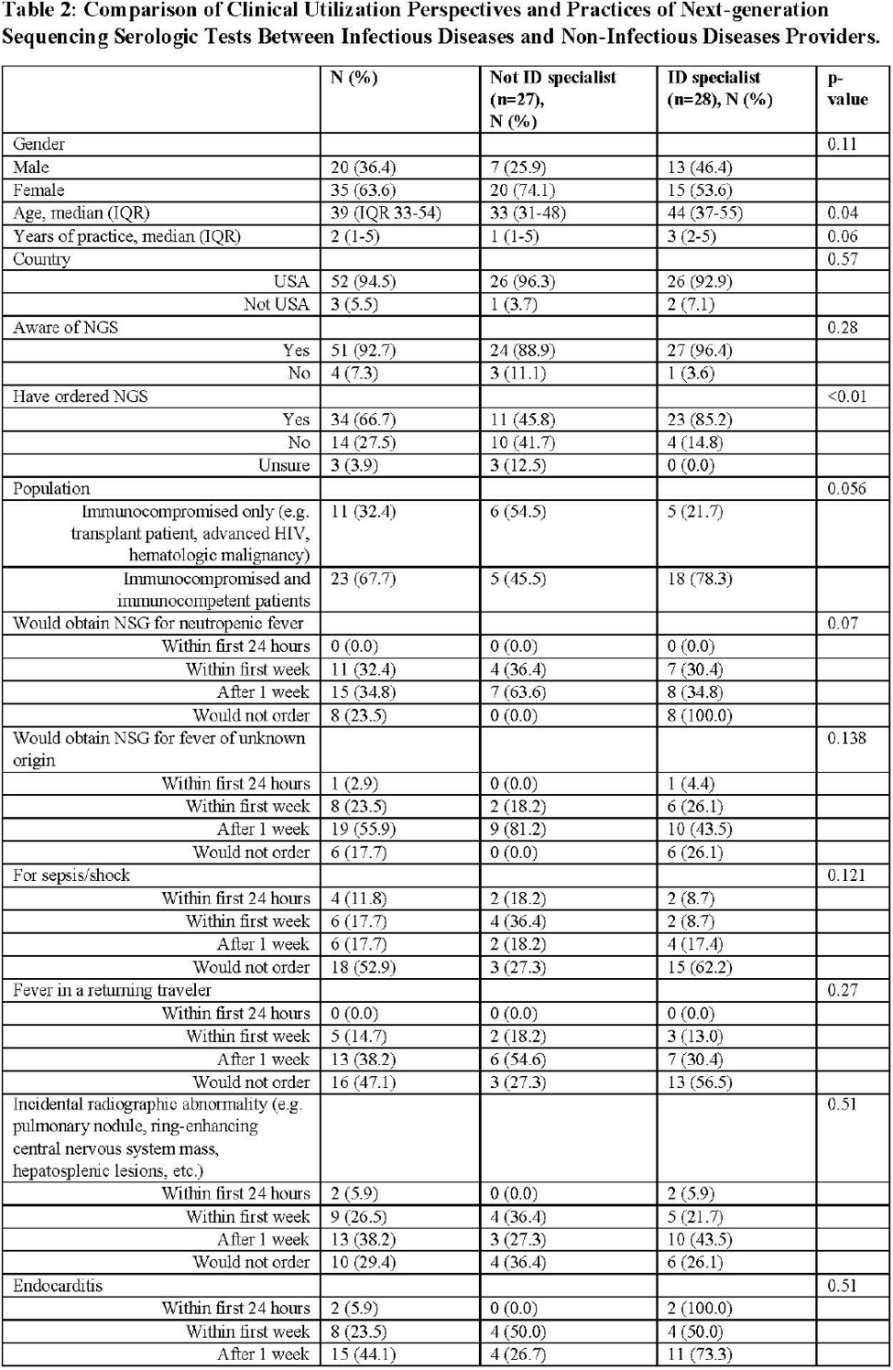
Comparison of Clinical Utilization Perspectives and Practices of Next-generation Sequencing Serologic Tests Between Infectious Diseases and Non-Infectious Diseases Providers

Page 1

**Table 2:**
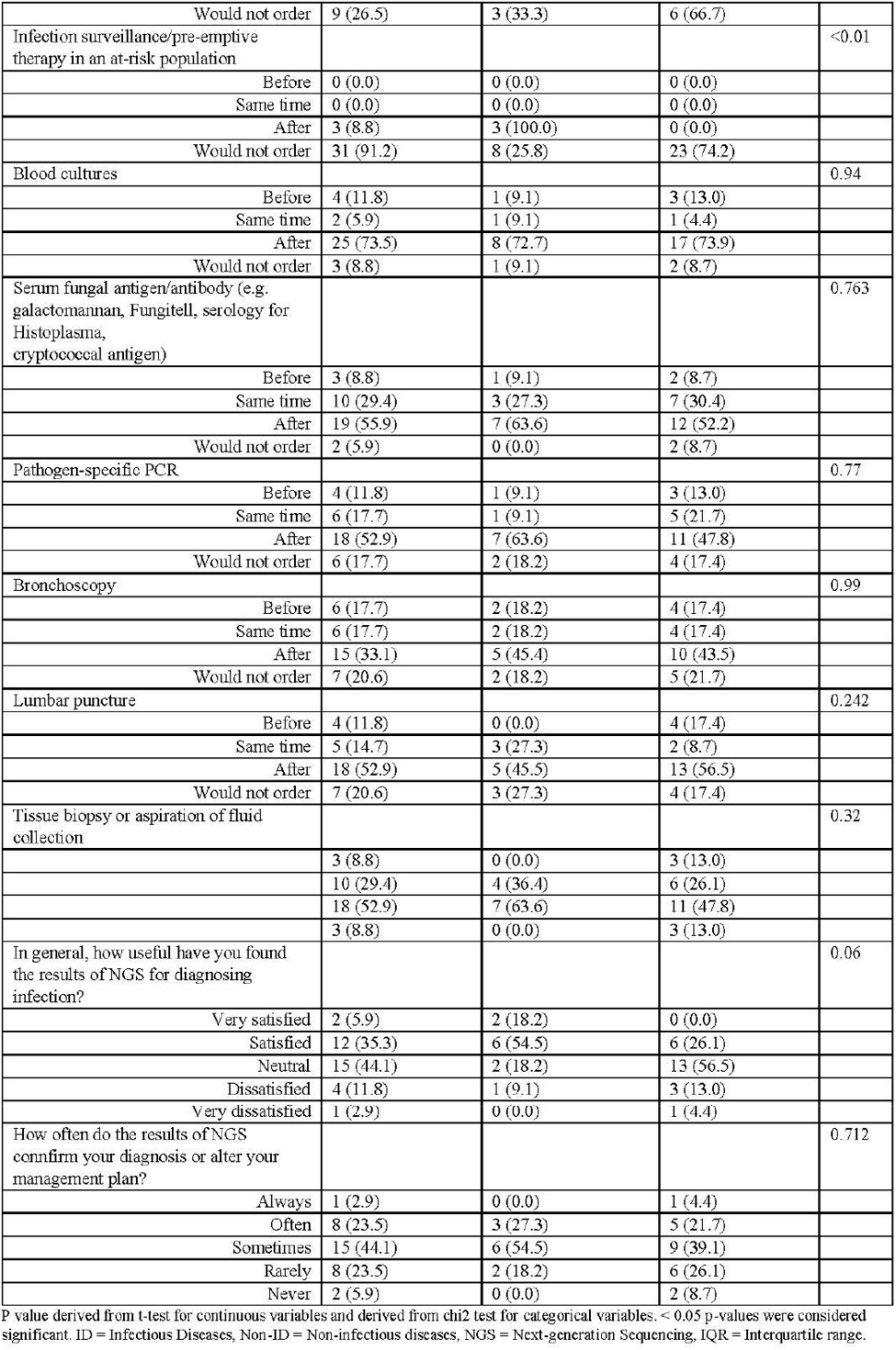
Comparison of Clinical Utilization Perspectives and Practices of Next-generation Sequencing Serologic Tests Between Infectious Diseases and Non-Infectious Diseases Providers

Page 2

**Results:**

55 participants initiated the survey, and 34 reported using NGS and completed the remainder of the survey. There most common specialties represented were adult ID (33%), pediatric hematology-oncology (33%), and pediatric ID (18%). The median age was 39 years (IQR 33-54), and 63.6% were female. 81.8% participants were from academic centers (Table 1). Compared to Non-ID providers, ID specialists were older (Median age 33 v. 44, p < 0.04) more likely to have ordered NGS in the past (85% v. 46%, p-value < 0.01), and less likely to order for infection surveillance/pre-emptive therapy in an at-risk population (0% v. 8.8%, p-value < 0.01; Table 2).

**Conclusion:**

Our cross-sectional survey revealed notable differences among providers regarding specific applications of NGS in clinical care. Although limited by a low response rate and survey completion percentage, this preliminary data could inform the development of enhanced NGS assessment projects. These insights may illuminate the use of NGS and influence future implementation and cost-effectiveness studies.

**Disclosures:**

All Authors: No reported disclosures

